# PD-L1^+^ neutrophils as novel biomarkers for stage IV melanoma patients treated with nivolumab

**DOI:** 10.3389/fimmu.2022.962669

**Published:** 2022-08-09

**Authors:** Leonardo Cristinziano, Luca Modestino, Mariaelena Capone, Gabriele Madonna, Domenico Mallardo, Diana Giannarelli, Grazia D’Angelo, Anne Lise Ferrara, Stefania Loffredo, Gilda Varricchi, Vito Vanella, Lucia Festino, Paolo Antonio Ascierto, Maria Rosaria Galdiero

**Affiliations:** ^1^ Department of Translational Medical Sciences (DiSMeT), University of Naples Federico II, Naples, Italy; ^2^ Center for Basic and Clinical Immunology Research, Interdipartimental Center for basic and Clinical Immunology Research (CISI), University of Naples Federico II, Naples, Italy; ^3^ World Allergy Organization (WAO) Center of Excellence, University of Naples Federico II, Naples, Italy; ^4^ Melanoma, Cancer Immunotherapy, and Development Therapeutics Unit, Istituto Nazionale Tumori IRCCS Fondazione “G. Pascale”, Naples, Italy; ^5^ Clinical Trial Center, Biostatistics and Bioinformatics Unit, Scientific Direction, Istituti di Ricovero e Cura a Carattere Scientifico (IRCCS) Regina Elena National Cancer Institute, Rome, Italy; ^6^ Institute of Experimental Endocrinology and Oncology, Istituto per l'Endocrinologia e l'Oncologia Sperimentale (IEOS), National Research Council (CNR), Naples, Italy

**Keywords:** melanoma, checkpoint inhibitors, nivolumab, tumor-associated neutrophil, neutrophil plasticity

## Abstract

Melanoma displays a rising incidence, and the mortality associated with metastatic form remains high. Monoclonal antibodies that block programmed death (PD-1) and PD Ligand 1 (PD-L1) network have revolutionized the history of metastatic disease. PD-L1 is expressed on several immune cells and can be also expressed on human neutrophils (PMNs). The role of peripheral blood PMNs as predictive biomarkers in anti-PD-1 therapy of melanoma is largely unknown. In this study, we aimed to determine activation status and PD-L1 expression on human neutrophils as possible novel biomarkers in stage IV melanoma patients (MPs). We found that PMNs from MPs displayed an activated phenotype and increased PD-L1 levels compared to healthy controls (HCs). Patients with lower PD-L1^+^ PMN frequencies displayed better progression-free survival (PFS) and overall survival (OS) compared to patients with high PD-L1^+^ PMN frequencies. Multivariate analysis showed that PD-L1^+^ PMNs predicted patient outcome in BRAF wild type MP subgroup but not in BRAF mutated MPs. PD-L1^+^ PMN frequency emerges as a novel biomarker in stage IV BRAF wild type MPs undergoing anti-PD-1 immunotherapy. Our findings suggest further evaluation of the role of neutrophil subsets and their mediators in melanoma patients undergoing immunotherapy.

## Introduction

Melanoma is a significant global public health issue, with an increasing incidence over the last few decades (1). Immune checkpoint inhibitors (ICIs) blocking programmed death-1 (PD-1) (nivolumab and pembrolizumab) or cytotoxic T-lymphocyte antigen-4 (CTLA-4) (ipilimumab) revolutionized the treatment of patients with advanced melanoma (2–4). Indeed, if less than 25% of advanced melanoma patients (MPs) were alive at 1 year in 2005, over 50% were alive at 5 years in 2019 (3, 5). During the phase 3 clinical trials, both pembrolizumab, and nivolumab exhibited superior efficacy in treatment-naive MPs compared to ipilimumab, with a 5-year overall survival of 43% for pembrolizumab and 44% for nivolumab, compared to 26% for ipilimumab (2, 3, 6, 7). However, a percentage of MPs still fail to respond or progress after initial therapy with anti-PD-1 +/- anti-CTLA-4 (8, 9). There is great interest in identifying patient subgroups who will obtain sufficient incremental benefit from the anti-PD-1 monotherapy, thus enabling them to avoid the increased risk of immune-related adverse events associated with the combination therapy. Thus, the identification of predictive biomarkers is a priority to improve the management of patients in the final target of personalized immunotherapy (8, 9). Expression of PD-Ligand 1 (PD-L1) on tumor cells revealed limitations due to technical issues and sample availability and repeatability (9). Analysis of peripheral blood immune cells, which is minimally invasive and repeatable, appears to be a more feasible approach. Neutrophils (polymorphonuclear leukocytes; PMNs) play a pivotal role in the acute inflammatory response and in defending against extracellular microbes (10). PMNs are a heterogeneous population endowed with surprising plasticity (11). Indeed, under the influence of different stimuli in the tumor microenvironment (TME), they can be polarized toward a pro-tumor or an anti-tumor phenotype (12). Increased densities of tumor-associated neutrophils (TANs) were significantly associated with patient prognosis in different human cancer types (13–16). Increased neutrophil infiltration of human lung and breast cancer predicted ICI treatment failure (17, 18). Moreover, elevated serum levels of the neutrophil-related cytokine IL-8 predicted the response to ICIs in melanoma, lung and renal cancer (19). PD-L1 positive PMNs have been associated with myriad immunologic disorders (20). To the best of our knowledge, the role of neutrophils as possible biomerkers of disease activity and anti-PD-1 therapy response in MPs is yet to be investigated. In this study, we performed a basal and longitudinal assessment of peripheral blood PMNs, from MPs treated with the anti-PD-1 monoclonal antibody nivolumab in a single-center cohort. The aim of our work was to evaluate the characteristics of the neutrophils from peripheral blood of advanced MPs under immunotherapy (anti-PD-1, nivolumab), to evaluate whether PMNs correlated with disease progression and response to therapy.

## Materials and methods

### Patients, treatment, and assessment

We built an observational cohort study by prospectively recruiting patients with stage IV melanoma candidates for PD-1 inhibitors (i.e., nivolumab). A total of 65 patients were recruited with a diagnosis of stage IV melanoma, according to the VII edition of American Joint Committee on Cancer (21) at the Istituto Nazionale Tumori—IRCCS—Fondazione “G. Pascale” of Naples, Italy. Response to therapy was evaluated according to RECIST V.1.1 criteria (22). All patients had provided written informed consent for the use of samples in accordance with the institutional regulations. Patients’ characteristics, including sex, age, distant metastasis, LDH serum levels, absolute neutrophil count (ANC), the status of *BRAF* mutation, and line of therapy are summarized in **Supplementary Table 1**.

Patients were treated with nivolumab at the standard dose (3 mg/kg every 2 weeks) or with the flat dosage of 240 mg every 2 weeks or 480 mg every 4 weeks. Treatment was continued until disease progression or the development of unacceptable toxic events. Radiological (MRI or CT scans of brain, bone, chest, abdomen, pelvis and other soft tissue as applicable) and visual (skin lesion) tumor assessments were undertaken at baseline and every 12 weeks, until progression or the discontinuation of therapy according to the Response Evaluation Criteria In Solid Tumors (RECIST) (version 1.1.). Peripheral blood samples were collected from all patients and freshly processed at baseline (before starting the therapy, on the day of the first cycle) and every 12 weeks. Overall survival (OS) was defined as the time from baseline visit (day 0 of treatment) to the last date of follow-up or to death from any cause. Progression-free survival (PFS) was the time from baseline visit to documented disease progression or death. Moreover, blood samples of 42 healthy donors, sex and age-matched, were collected at the University of Naples Federico II, Naples, Italy. The study was approved by the local Ethics Committee of the Istituto Nazionale Tumori - IRCCS - Fondazione “G. Pascale” of Naples (prot. no 33/17) and University of Naples Federico II (n. 301/18) and was conducted in compliance with the international standards of good clinical practice. The study was conducted in accordance with the provisions of the Declaration of Helsinki.

### Flow cytometry analysis

Blood samples (20 mL) were collected from all MPs into EDTA vacutainer (Becton Dickinson, NJ USA) and PMNs were isolated within 2 hours of blood collection. Peripheral blood leukocytes were isolated from erythrocytes by 3% dextran sedimentation (PanReac AppliChem ITW Reagents, Darmstadt, Germany) before being washed in phosphate-buffered saline (PBS). PMNs were freshly isolated by density centrifugation (400 × g for 30 minutes at 22°C) using Ficoll^®^ Paque Plus (GE17-1440-02, Sigma, St. Louis, MO, USA). After centrifugation, PMNs were further purified by 65% Percoll (Sigma Aldrich, Milan, Italy) density gradient centrifugation (660 × g for 20 minutes at 22°C) (23). Zombie Violet dye (Biolegend, CA, USA) was used to evaluate cell viability. After 20 minutes of incubation, PMNs were washed with PBS and staining was performed. The following antibodies were used: Allophycocyanin (APC)-conjugated anti-CD66b (1:50, from Miltenyi Biotec, Bergisch Gladbach, Germany), peridinin chlorophyll protein (PerCP)-conjugated anti-CD11b (1:50, from Miltenyi Biotec, Bergisch Gladbach, Germany), VioBlue-conjugated anti-CD193 (1:10, from Miltenyi Biotec, Bergisch Gladbach, Germany), fluorescein isothiocyanate (FITC)-conjugated anti-CD62L (1:50, from Miltenyi Biotec, Bergisch Gladbach, Germany), phycoerythrin (PE)-conjugated anti-PD-L1 (1:10, from Biolegend, CA, USA), phycoerythrin (PE)-conjugated anti-CD16 (1:50, from Miltenyi Biotec, Bergisch Gladbach, Germany). The cells were washed and analyzed using the MACS Quant Analyzer 10 (Miltenyi Biotec, Bergisch Gladbach, Germany) and the FlowJo software, v.10 after 20 minutes of incubation. Doublets and debris were identified based on forward- and side-scatter properties and excluded from the analysis. Dead cells and eosinophils were also excluded with a CCR3/Zombie Violet die negative gating strategy. Fluorescence minus one (FMO) controls, internal negative controls, and compensation controls were used, as recommended by the literature to validate flow cytometry multicolor panels (24). Data were expressed as percentage of PD-L1 positive cells compared to FMO controls, gated on CCR3^-^CD66b^+^CD11b^+^ neutrophils (25). A complete example of the gating strategy is represented in **Supplementary Figure 1**.

### Statistical analysis

Results are presented using absolute frequencies and percentages when referring to categorical variables, and mean ± SD when considering quantitative variables. Patient baseline characteristics were described using descriptive statistics. Due to the parametric distribution of the variables, differences in cells subsets’ frequencies between patients and controls or between patient subgroups were evaluated with the Student t test. PFS time was calculated from the date of the first dose of nivolumab to the date of progression or death, whichever occurs first; OS was calculated from the date of first dose of nivolumab to the date of death. Both times were censored at the date of the last follow-up. The cut-off score was selected based on the median value. Survival time was analyzed with the Kaplan-Meier method, and the log-rank test was used to test for differences. The R-package ‘survival’(version 3.2, published by Terry M. Therneau and Thomas Lumley on 2021-04-26; https://CRAN.R-project.org/package=survival) and ‘survminer’ (version 0.4.9 by Alboukadel Kassambara, Marcin Kosinski, Przemyslaw Biecek published 2021-03-09; https://CRAN.R-project.org/package=survminer) were applied for univariate and multivariate Cox proportional hazard models to calculate HR and 95% CI of different variables, including sex, age, distant metastasis, LDH serum levels, ANC, the status of *BRAF* mutation, line of therapy, and basal % PD-L1^+^ PMNs. Likehood and Wald tests were used to evaluate model validity (*p* values ≤ 0.05). Furthermore, the interactions and subgroup analysis were assessed for *p* values ≤ 0.05. According to the RECIST disease classification (26), patient clinical responses were classified into SD (stable disease), PR (partial response), CR (complete response), and PD (progressive disease). The overall response rate (ORR) to nivolumab was calculated as the percentage of patients with CR + the percentage of patients PR. The disease control rate (DCR) to nivolumab was calculated as the percentage of patients with CR + the percentage of patients with PR and with SD. The associations between pretreatment levels of PD-L1^+^ PMNs, ORR, and DCR were analyzed by the Fisher’s exact test or chi-squared test. The analyses were performed using GraphPad Prism software (version 8.0, San Diego, CA, USA) and R Studio (version 4.0.5, Boston, MA, USA), a language and statistical computing{Team, #53}{Team, #53}. Mean ± SD is shown in the figures. For all the analyses, statistical significance was set at *p*<0.05.

## Results

### Demographic and clinical characteristics of melanoma patients

This study included a total of 65 patients with stage IV melanoma. The baseline clinic-pathological characteristics of all patients are summarized in **Supplementary Table 1**. The median age was 61 years old; 30 patients (46.2%) were male and 35 (53.8%) were female. Twenty-two patients had a melanoma harboring *BRAF* mutation and 39 patients were *BRAF* wild-type; in 4 patients, the *BRAF* mutational status was unknown. All patients received PD-1 monotherapy with nivolumab. As for the line of therapy, 43 patients received nivolumab as first-line, 22 patients received nivolumab as second-line therapy or later. According to the site of distant metastasis and LDH levels (27) patients were distributed as follows: 5/65 (7.7%) patients as M1a; 9/65 (13.8%) patients as M1b, 31/65 (47.7%) patients as M1c and 20/65 (30.8%) patients as M1d. 32 patients (50%) displayed circulating levels of LDH within the normal range, and 32 patients (50%) displayed circulating LDH levels upper the normal range (one LDH value was missed). A total of 17 patients (20%) had a complete response (CR, n=9) or partial response (PR, n=8), while the others (65%) displayed progressive disease (PD, n=29) or stable disease (SD, n=19). The median PFS was 8.97 months (IQR: 2.4 to 27.83) and the median OS was 15.17 months (IQR: 4.97 to 34.13).

### Frequencies and activation status of PMNs in melanoma patients and healthy controls

**Figure 1:**
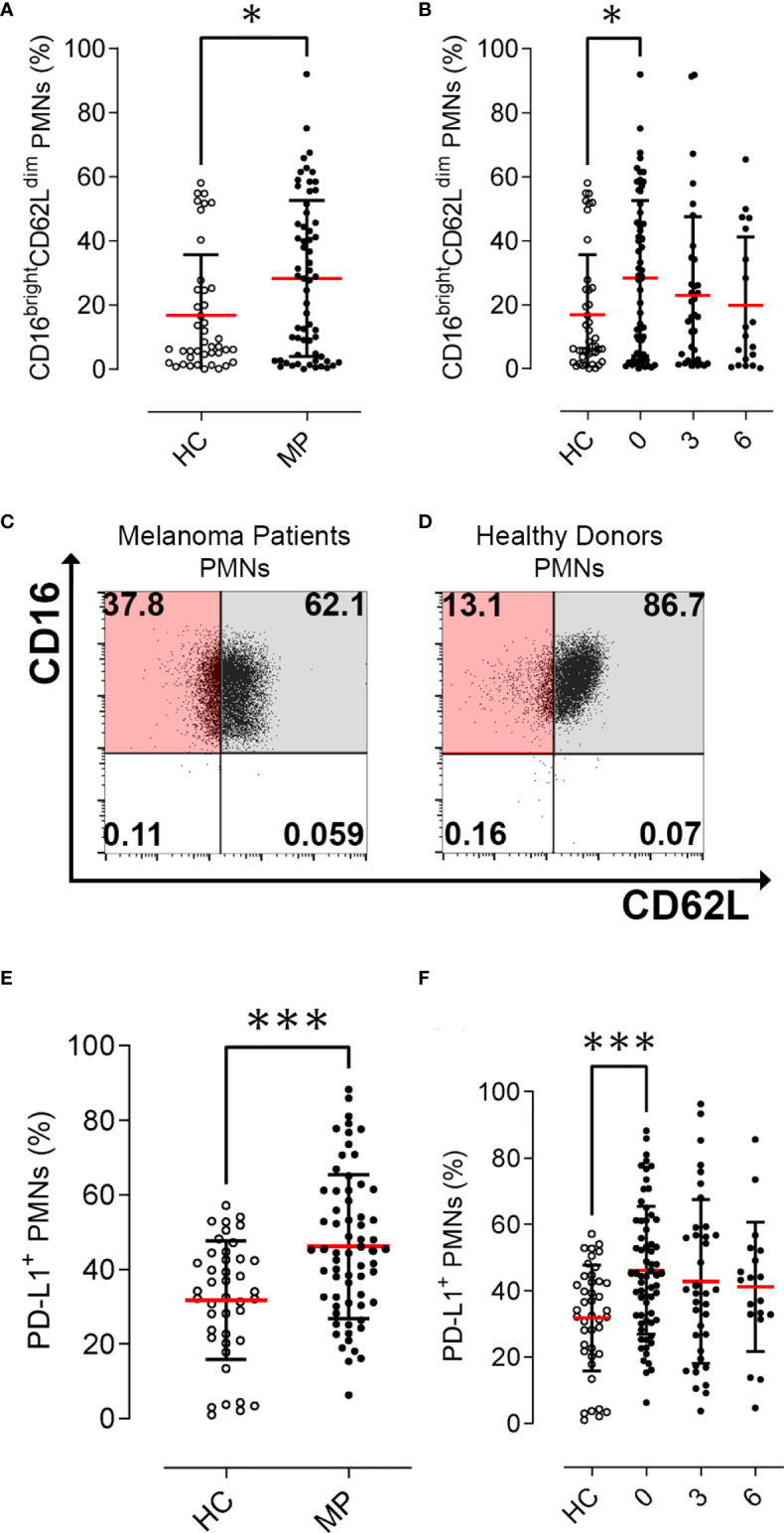
Activation status and PD-L1^+^ PMN frequencies in melanoma patients (MPs; filled dots) and healthy controls (HCs; open dots). Flow cytometry analysis of % CD16^bright^ CD62L^dim^ cells gated on PMNs at baseline **(A)** and during nivolumab immunotherapy **(B)**. Representative flow cytometric panels illustrating scatter plot of CD16^bright^ CD62L^dim^ cells in PMNs in melanoma patients **(C)** and healthy controls **(D)**. Flow cytometry analysis of PD-L1^+^ live cells gated on CD66b^+^ CD11b^+^PMNs at baseline **(E)** and during nivolumab immunotherapy **(F)**. Data were expressed as percentage of PD-L1 positive cells compared to FMO controls, gated on CCR3^-^CD66b^+^CD11b^+^ neutrophils. The results were expressed as mean ± SD. * p<0.05; *** *p* < 0.001. Student’s T test.

We prospectively investigated the frequency of peripheral blood PMNs by means of flow cytometry analysis. The characterization of these cells was performed on freshly obtained blood samples from MPs before starting nivolumab therapy and every 12 weeks of therapy. In parallel experiments, we prospectively analyzed PMNs from healthy controls (HCs). The mean absolute number of peripheral blood neutrophils (ANC) of melanoma patients (MPs) was found to be within the normal range (5044 cells/μl ±3441 cells/μl). To evaluate the activation status of human PMNs isolated from peripheral blood of MPs and HCs, we determined CD16 and CD62L (L-selectin) expression by flow cytometry (28, 29). PMNs from MPs and HCs were stained with antibodies against CD16 and CD62L and evaluated by flow cytometry (15). Under resting conditions, neutrophils express CD62L, which rapidly decreases (i.e., shedding) after activation. Indeed, the CD16^bright^/CD62L^dim^ cells consist mainly of neutrophils containing hypersegmented nuclei suggesting a more activated state (30). MPs displayed increased frequencies of activated CD16^bright^CD62L^dim^ PMNs compared with HCs (28.3% *vs.* 16.9%, *p*=0.02, (**Figure 1A**). The frequency of CD16^bright^CD62L^dim^ PMNs tended to decrease during immunotherapy without reaching statistical significance (28.3% at baseline, 23% at 3 months, p=0.27; 19.9% at 6 months, p=0.20, (**Figure 1B**). Collectively, these data indicate that peripheral blood PMNs from MPs displayed an activated status (CD16^bright^CD62L^dim^) in comparison to PMNs from HCs, which was not modified by immunotherapy. Representative flow cytometric panels illustrating scatter plot of CD16^bright^ CD62L^dim^ cells in PMNs in MPs and HCs are illustrated in (**Figures 1C, D**).

### PD-L1^+^ PMN frequencies and patient survival

We prospectively investigated the frequency of peripheral blood PMNs positive to PD-L1 (PD-L1^+^ PMNs) by flow cytometry analysis. The characterization of these cells was performed on freshly obtained blood samples from MPs before commencing nivolumab therapy and every 12 weeks of therapy. PD-L1^+^ PMNs frequency was increased in MPs compared with HCs (46.1% *vs.* 31.8%, *p*=0.0001, **Figure 1E**). PD-L1^+^ PMNs levels showed a slight but not significant decrease during immunotherapy (46.1% at baseline, 42.7% at 3 months; p=0.44; 41.1% at 6 months; p=0.33, (**Figure 1F**). We then investigated the correlations between PD-L1^+^ PMNs and CD16^bright^CD62L^dim^ PMNs. No significant correlations were found between PD-L1 expression and activation status in PMNs (data not shown). We then analyzed the distributions of the mean frequencies of PD-L1^+^ PMNs in relationship to patient clinic-pathological features (i.e. age, gender, presence of *BRAF* mutation, line of therapy, distant metastasis, LDH serum levels, and ANC) (**Supplementary Table 2**). PD-L1^+^ PMN frequencies were not associated with any patient clinic-pathological feature. No significant differences were found between patients with or without *BRAF* mutations or in relation to different sites of distant metastasis, LDH serum levels, ANC, or lines of therapy (**Supplementary Table 2**).

**Figure 2:**
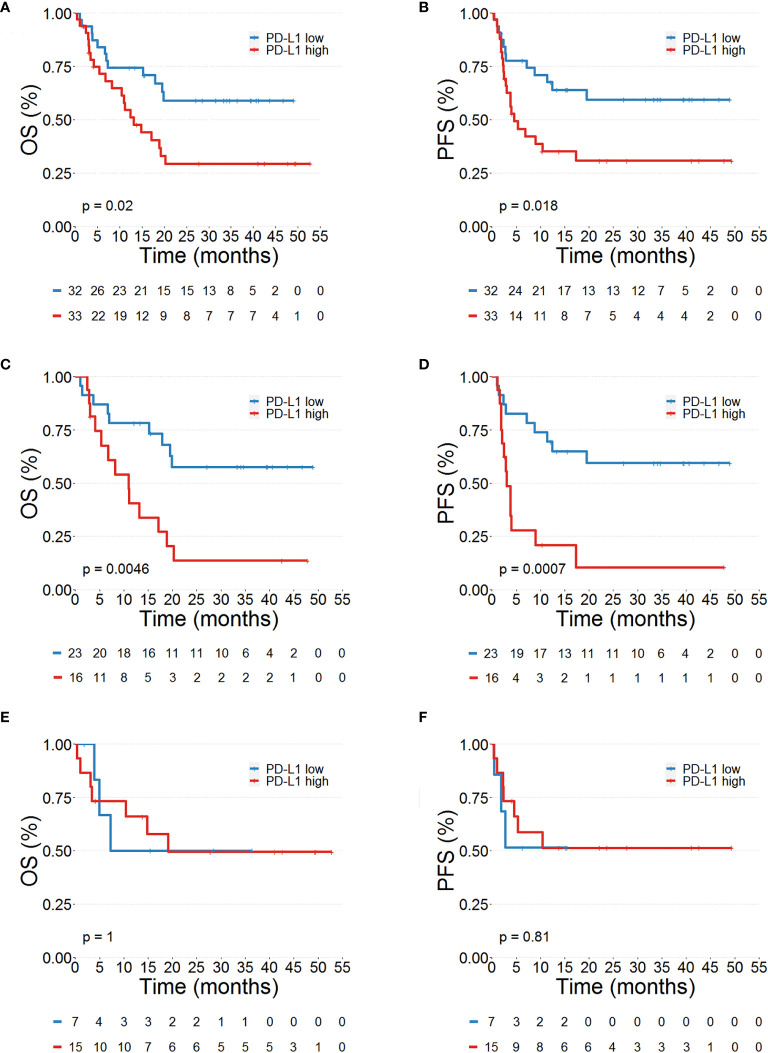
Prognostic significance of PD-L1^+^ neutrophils in advanced melanoma patients. Kaplan–Meier survival curves show overall survival (OS %) **(A, C, E)** and progression-free survival (PFS %) **(B, D, F)** for advanced melanoma patients presenting a high (red line) or low (blue line) PD-L1^+^ PMNs in the whole patient cohort **(A, B)**, in *BRAF* wild-type **(C, D)** and *BRAF* mutated **(E, F)** melanoma patients. Low and high PD-L1^+^ values were calculated using the median as the cut-off.

We then tested the association between PD-L1^+^ PMNs, CD16^bright^CD62L^dim^ PMNs and OS and PFS. The median value of each variable was used to identify patient subgroups. Kaplan–Meier curves of OS and PFS were plotted and the log rank test was used to compare the curves of patient subgroups. Interestingly, a high PD-L1^+^ PMN frequency was correlated with adverse OS (*p*=0.02, **Figure 2A**) and PFS (*p*=0.018, **Figure 2B**). No difference in OS and PFS between MPs with high and low CD16^bright^CD62L^dim^ PMNs were found (data not shown). Collectively, these results suggest that PD-L1^+^ PMN frequency could represent a circulating biomarker predicting MP prognosis.

### PD-L1^+^PMN frequencies as novel prognostic biomarkers in *BRAF* wild type melanoma patients

The frequency of peripheral blood PD-L1^+^ PMNs in MPs together with clinical characteristics were analyzed to identify pre-treatment factors associated with OS and PFS by Cox regression analysis (**Table 1**). Median values of PD-L1^+^ PMN frequencies were used to divide patients into high and low PD-L1^+^ PMNs. Values were entered into a Cox proportional hazard model to evaluate their potential impact on melanoma outcome in addition to the clinic-pathological features. Coherently, at univariate analysis, patients undergoing nivolumab as second/third line of therapy displayed worse outcomes compared with patients undergoing nivolumab as the first line of therapy (*p*=0.016 and HR=2.33 for OS; *p*=0.0083 and HR= 2.35 for PFS, **Table 1**). In addition, higher circulating levels of LDH were associated with worse patient OS (p=0.0019; HR= 3.11) and PFS (p=0.0029; HR= 2.65). ANC was also associated with worse patient OS (p=0.000079; HR= 1.02) and PFS (p=0.0045; HR= 1.01). Moreover, in the model, high PD-L1^+^ PMN frequency was significantly associated with worse outcomes (*p*=0.026 for OS and *p*=0.013 for PFS, **Table 1**). Results were paralleled by Kaplan–Meier curves, showing a worse outcome in patients with higher frequencies of PD-L1^+^ PMNs (**Figure 2A**).We incorporated frequencies of PD-L1^+^ PMNs in a multivariate Cox proportional hazard model to assess whether the detected potential predictive value of PD-L1^+^ PMNs could be influenced by other variables. Multivariate analysis revealed that PD-L1^+^ PMNs frequency was not associated with a likelihood of different outcomes (*p*=0.14 for OS and *p*=0.25 for PFS, **Table 1**). However, we found a statistical interaction between PD-L1^+^ PMN frequency and *BRAF* status (*p*=0.01), suggesting that the predictive significance of PD-L1^+^ PMN frequencies could be modified in the different settings of this parameter (*BRAF* mutated *vs.* wild-type melanoma) across the study cohort (**Table 1**). Subsequently, patients were distributed in two different subgroups, namely with or without *BRAF* mutations. We then tested the association between PD-L1^+^ PMNs and OS and PFS in the two different patient subgroups. Kaplan–Meier curves of OS and PFS were plotted and the log rank test was used to compare the curves of patient subgroups. A high PD-L1^+^ PMN frequency was correlated with adverse OS and PFS in MPs without *BRAF* mutations (**Figures 2C, D**). By contrast, in *BRAF* mutated MP subgroup, no differences were found between patients with high and low PD-L1^+^ PMN frequencies on OS and PFS (**Figures 2E, F**). As illustrated in **Table 2**, among *BRAF* wild type MPs, patients with higher PD-L1^+^ PMN frequencies displayed a worse OS (*p*=0.0072, HR= 3.27) and PFS (*p*=0.00026; HR= 5.01) at univariate analyses. Upon multivariate analyses, patients with higher PD-L1^+^ PMNs presented a hazard ratio of 3.2 and 4.84 respectively for OS and PFS, compared with patients with lower PD-L1^+^ PMNs and the results were independent of all other variables (*p*=0.027 for OS and *p*=0.002 for PFS, **Table 2**). Congruently, the subgroup analysis restricted to *BRAF* wild type melanoma patients showed that PD-L1^+^ PMN frequency emerges as an independent predictive factor in this patient subgroup selectively.

**Table 1:** Univariate and multivariate analyses for OS and PFS in advanced melanoma patients.

		OS						PFS					
		Univariate analysis			Multivariate analysis			Univariate analysis			Multivariate analysis		
	N	HR	95% CI	*p* value	HR	95% CI	*p* value	HR	95% CI	*p* value	HR	95% CI	*p* value
**Age (years)**													
<61	32	1.00 Ref.			1.00 Ref.			1.00 Ref.			1.00 Ref.		
≥61	33	1.19	(0.6-2.4)	0.62	1.23	(0.54-2.81)	0.61	1.22	(0.65-2.3)	0.54	1.17	(0.52-2.61)	0.70
**Gender**													
Male	30	1.00 Ref.			1.00 Ref.			1.00 Ref.			1.00 Ref.		
Female	35	0.95	(0.48-1.9)	0.89	0.78	(0.35-1.73)	0.54	1.13	(0.6-2.1)	0.7	0.91	(0.43-1.89)	0.80
***BRAF* mutation**													
no	39	1.00 Ref.			1.00 Ref.			1.00 Ref.			1.00 Ref.		
yes	22	0.89	(0.42-1.9)	0.76	0.48	(0.19-1.19)	0.11	1.15	(0.59-2.2)	0.68	0.69	(0.29-1.63)	0.39
**Line of treatment**													
1	43	1.00 Ref.			1.00 Ref.			1.00 Ref.			1.00 Ref.		
2 and 3	22	2.33	(1.2-4.6)	**0.016**	1.90	(0.80-4.54)	0.15	2.35	(1.2-4.4)	**0.0083**	1.86	(0.85-4.07)	0.12
**Distant metastasis**													
M1a/M1b/M1c	45	1.00 Ref.			1.00 Ref.			1.00 Ref.			1.00 Ref.		
M1d	20	1.87	(0.92-3.8)	0.086	1.34	(0.60-2.95)	0.47	2.00	(1.1-3.8)	**0.032**	1.55	(0.75-3.20)	0.23
**LDH**													
Normal	32	1.00 Ref.			1.00 Ref.			1.00 Ref.			1.00 Ref.		
Upper limit of normal	32	3.11	(1.5-6.4)	**0.0019**	2.48	(1.08-5.70)	**0.03**	2.65	(1.4-5)	**0.0029**	2.04	(0.96-4.37)	0.06
**ANC ^1^**	58	1.02	(1-1)	**0.000079**	1.02	(1-1.03)	**0.001**	1.01	(1-1)	**0.0045**	1.01	(1-1.02)	0.06
**Basal % PD-L1+ PMNs**													
< 44.9	32	1.00 Ref.			1.00 Ref.			1.00 Ref.			1.00 Ref.		
≥ 44.9	33	2.24	(1.1-4.6)	**0.026**	1.94	(0.80-4.65)	0.14	2.27	(1.2-4.3)	**0.013**	1.60	(0.72-3.53)	0.25 **^2^**

**^1^** Absolute neutrophil count (ANC) entered as a continuous variable.

**^2^** Interaction between PD-L1 and BRAF, p= **0.01**.

**N**, number; **HR**, hazard ratio; **CI**, confidence interval; **OS**, overall survival; **PFS**, progression-free survival; **ANC**, absolute neutrophil count; **PMNs**, polymorphonuclear cells.Statistically significant results were reported in bold.

**Table 2:** Univariate and multivariate analyses for OS and PFS in *BRAF* Wild type advanced melanoma patients.

		OS						PFS					
		Univariate analysis			Multivariate analysis			Univariate analysis			Multivariate analysis		
	N	HR	95% CI	*p* value	HR	95% CI	*p* value	HR	95% CI	*p* value	HR	95% CI	*p* value
**Age (years)**													
<61	13	1.00 Ref.			1.00 Ref.			1.00 Ref.			1.00 Ref.		
≥61	26	1.51	(0.61-3.7)	0.37	1.87	(0.67-5.24)	0.23	1.30	(0.54-3.1)	0.56	1.30	(0.45-3.80)	0.63
**Gender**													
Male	21	1.00 Ref.			1.00 Ref.			1.00 Ref.			1.00 Ref.		
Female	18	1.05	(0.46-2.4)	0.9	0.73	(0.28-1.94)	0.53	1.10	(0.49-2.4)	0.82	0.49	(0.17-1.37)	0.17
***BRAF* mutation**													
no		NA			NA			NA			NA		
yes		NA			NA			NA			NA		
**Line of treatment**													
1	32	1.00 Ref.			1.00 Ref.			1.00 Ref.			1.00 Ref.		
2 and 3	7	2.04	(0.82-5.1)	0.13	1.10	(0.35-3.55)	0.86	2.19	(0.9-5.3)	0.083	1.26	(0.40-3.97)	0.70
**Distant metastasis**													
M1a/M1b/M1c	27	1.00 Ref.			1.00 Ref.			1.00 Ref.			1.00 Ref.		
M1d	12	1.50	(0.63-3.6)	0.36	1.66	(0.57-4.86)	0.35	1.41	(0.62-3.2)	0.42	1.15	(0.40-3.33)	0.80
**LDH**													
Normal	23	1.00 Ref.			1.00 Ref.			1.00 Ref.			1.00 Ref.		
Upper limit of normal	15	2.25	(0.97-5.2)	0.059	1.49	(0.52-4.28)	0.46	2.55	(1.1-5.7)	**0.023**	2.18	(0.71-6.71)	0.17
**ANC ^1^**	58	1.01	(0.99-1)	0.21	1.01	(1-1.03)	0.27	1.01	(0.99-1)	0.49	1.01	(0.97-1.01)	0.67
**Basal % PD-L1+ PMNs**													
<44.9	23	1.00 Ref.			1.00 Ref.			1.00 Ref.			1.00 Ref.		
≥ 44.9	16	3.27	(1.4-7.8)	**0.0072**	3.20	(1.14-9)	**0.027**	5.01	(2.1-12)	**0.00026**	4.84	(1.76-13.3)	**0.002**

**^1^** Absolute neutrophil count (ANC) entered as a continuous variable.

**N**, number; **HR**, hazard ratio; **CI**, confidence interval; **OS**, overall survival; **PFS**, progression-free survival; **ANC**, absolute neutrophil count; **PMNs**, polymorphonuclear cells.Statistically significant results were reported in bold.NA, Not Available.

### PD-L1^+^PMN frequency and response to therapy in *BRAF* wild type melanoma patients

Given that our data indicated an association between the frequency of PD-L1^+^ PMNs and survival, we investigated correlations between the frequencies of these cells with ORR and DCR to nivolumab. According to the RECIST disease classification (26), patient clinical responses were classified into SD, PR, CR, and PD. ORR to nivolumab was 26% (17/65: 9 patients with CR, 8 patients with PR). DCR to nivolumab was 55% (36/65: 9 patients CR, 8 patients with PR, and 19 with SD). A total of 29 patients showed PD. The pretreatment frequencies of PD-L1^+^ PMNs were significantly associated with ORR (*p*=0.04) and DCR (*p*=0.03, **Table 3**). Indeed, a higher percentage of patients with CR or PR presented lower frequencies of PD-L1^+^ PMNs in comparison to patients with higher PD-L1^+^ PMNs frequencies (37.5% *vs.* 15.2%; *p*=0.04, **Table 3**). Moreover, a higher percentage of patients with DCR presented lower frequencies of PD-L1^+^ PMNs (68.8% *vs.* 42.4%; *p*=0.03, **Table 3**). In addition, we examined the association between PD-L1^+^ PMNs and clinical response in two patient subgroups, namely with or without *BRAF* mutations. Both ORR and DCR were associated with PD-L1^+^ PMN frequencies in MPs without *BRAF* mutations (*p*=0.02 and *p*=0.001, **Table 3**). By contrast, in the *BRAF* mutated MP subgroup, PD-L1^+^ PMN frequencies failed to predict patient ORR to nivolumab (*p*=0.52, **Table 3**) and were inversely associated with DCR (28.6% responders with low PD-L1^+^ NDNs *vs.* 66.7% responders with high PD-L1^+^ PMNs, *p*=0.04, **Table 3**). According to these data, pre-treatment PD-L1^+^ PMN frequencies selectively predict patient clinical outcome and therapeutic response to nivolumab in melanoma patients without *BRAF* mutations.

**Table 3:** Correlations between PD-L1^+^ PMN frequencies and clinical response in advanced melanoma patients.

	ORR	*p* value	DCR	*p* value
**All Patients**				
PD-L1^+^ PMNs				
< 44.9	12/32 (37.5%)	**0.04**	22/32 (68.8%)	**0.03**
≥ 44.9	5/33 (15.2%)		14/33 (42.4%)	
***BRAF* mutation**				
**NO**				
PD-L1^+^ PMNs				
< 44.9	9/23 (39.1%)	**0.02**	18/23 (78.3%)	**0.001**
≥ 44.9	1/16 (6.2%)		4/16 (25.0%)	
**YES**				
PD-L1^+^ PMNs				
< 44.9	1/7 (14,3%)	0.52	2/7 (28,6%)	**0.04**
≥ 44.9	4/15 (26.7%)		10/15 (66.7%)	

**ORR**, overall response rate; **DCR**, disease control rate; **PMNs**, polymorphonuclear cells. p value obtained in the Chi-square test.Statistically significant results were reported in bold.

## Discussion

Despite a rising incidence, likely due to the improvement in early diagnosis, the overall survival of advanced MPs significantly improved in the last 5 years (1). Indeed, the introduction of immunotherapy has revolutionized the therapeutic approach to melanoma and monoclonal antibodies targeting the PD-1/PD-L1 axis have significantly prolonged patient survival (2–5, 31). However, there is great interest in identifying patient subgroups who are most likely to benefit from the anti-PD-1 immunotherapy since a proportion of patients still fail to respond or progress after initial therapy (8, 9). In this study, we analyzed the frequencies of peripheral blood PD-L1^+^ neutrophil subsets in advanced MPs before and during nivolumab treatment. According to our findings, advanced MPs displayed a higher basal frequency of PD-L1^+^ PMNs compared to HCs, which was not modified during immunotherapy. MPs also displayed increased frequencies of activated CD16^bright^CD62L^dim^ PMNs compared with HCs. The frequencies of CD16^bright^CD62L^dim^ PMNs tended to decrease during immunotherapy without reaching statistical significance. Moreover, we found that frequencies of PD-L1^+^ PMNs selectively predicted patient response to nivolumab in *BRAF* wild type MPs. These results suggest that peripheral blood PD-L1^+^ PMN frequency could represent a novel biomarker of *BRAF* wild type MPs. Anti-PD-1 monoclonal antibodies, by neutralizing T cell inactivation in the TME, promote an anti-tumor response in different types of cancers, thus improving patient survival (32). However, a proportion of patients still do not respond or progress after initial therapy (3), underscoring the need to delve deeper into the immunological mechanisms responsible for this lack of response. With this in mind, the identification of biomarkers useful to efficiently predict patient therapeutic response to these biological agents could avoid unnecessary toxicity and wasted treatment resources. To date, some factors that correlate with clinical response to anti-PD-1 agents have been identified. Moreover, high eosinophil count, lymphocyte count, low LDH, and absence of metastasis other than soft-tissue/lung have been associated with better survival in MPs treated with anti-PD-1 agents (33, 34). A growing body of evidence suggests that neutrophils play a pivotal role in cancer-related inflammation (15, 16, 35). Neutrophil-to-lymphocyte ratio (NLR) has been proposed, as a prognostic marker in solid tumors, including melanoma (36). Baseline NLR was proposed as an independent predictive factor for response to ipilimumab and to nivolumab in melanoma patients (33, 37–39). Furthermore, it has been reported that high NLR at baseline independently predicted patient worse survival (33, 37–39). However, NLR is not a powerful predictive factor for several reasons. First, NRL is rather unspecific because several pathologic conditions are often associated with enhanced granulopoiesis (40, 41). Second, there is compelling evidence that human peripheral blood neutrophils comprise different subsets of cells with divergent functions (42, 43). Therefore, the NLR cannot identify qualitative changes in neutrophil subsets. In this study, we found increased baseline frequencies of peripheral blood PD-L1^+^ PMNs in advanced MPs compared to HCs, which were not modified during immunotherapy. An increasing number of studies are starting to highlight the significance of PD-L1^+^ neutrophils in cancer growth. For instance, in gastric cancer (GC), tumor-derived GM-CSF induced PD-L1 expression on neutrophils, *via* Jak-Stat3 signaling pathway, suppressing T-cell immunity and contributing to GC progression (44). In hepatocellular carcinoma (HCC), PD-L1^+^ neutrophils accumulate in the peritumoral regions and suppress the activation and proliferation of T cells (45). In addition, cancer-associated fibroblasts (CAFs) can induce PD-L1 expression on neutrophils to impair T-cell function by IL-6 - Stat3 signaling pathway (46). The frequency of PD-L1^+^ neutrophils in peripheral blood of patients with poorly differentiated HCC was significantly increased and independently predicted poor prognosis (47). In an *in vitro* model of human breast cancer, tumor-derived CCL20 activated and up-regulated PD-L1 expression on TANs, which suppressed T cells in a PD-L1-dependent manner (48). In line with these observations, our results show an increased PD-L1^+^ PMN frequencies suggesting potential tumor-promoting effects, which could at least partially account for the poor prognosis of advanced melanoma patients. We also investigated the neutrophil activation state in this study. Acute inflammation was associated with the rapid occurrence of a population of CD16^bright^/CD62L^dim^ neutrophils which can suppress T cell proliferation (30). The frequencies of CD16^bright^/CD62L^dim^ neutrophils were increased in peripheral blood of patients with solid tumors (49) and leukemia (50) and showed immunosuppressive properties *in vitro* (50). We found increased frequencies of CD16^bright^/CD62L^dim^ neutrophils in MPs compared to HCs. In our cohort, these cells were not associated with patient survival or clinical response.

*BRAF* mutations occur in about 40–60% of melanomas and the vast majority are observed within exon 15 (codon 600, namely *BRAF* ^V600E^) (51). *BRAF*-mutated melanomas are characterized by more aggressive clinical features compared to wild-type ones (51). Our results have unraveled a novel and potentially interesting association between PD-L1^+^ PMNs and *BRAF* status in melanoma patients. PD-L1^+^ PMN frequency was associated with worse patient survival only in *BRAF* wild type melanoma patients but not in *BRAF* mutated melanoma patients. This interesting observation indicates that, in advanced MPs, PD-L1^+^ PMN frequency might have a dual clinical significance, depending on the presence of *BRAF* mutation. Accordingly, we investigated the association between PD-L1^+^ PMNs and clinical response in the two patient subgroups, based on the results obtained with survival, namely *BRAF* wild type and *BRAF* mutated melanoma patients. Interestingly, both ORR and DCR were associated with PD-L1^+^ PMN levels in *BRAF* wild type melanoma patients. By contrast, in the *BRAF* mutated melanoma patient subgroup, PD-L1^+^ PMN frequencies failed to predict patient ORR to nivolumab whereas were inversely associated with DCR. These data indicate that pre-treatment PD-L1^+^ PMN frequencies can predict patient clinical outcome and therapeutic response to nivolumab only in *BRAF* wild type melanoma patients. Based on these findings, we also investigated the frequency of PD-L1^+^ PMNs during immunotherapy in MP grouped according to the status of *BRAF* (mutated versus wild type). As shown in the **Supplementary Figure 2**, in the *BRAF* wild type patient subgroup, the frequency of PD-L1^+^ PMNs tended to decrease during immunotherapy without reaching statistical significance (42.7% at baseline, 44.9% at 3 months; 35.6% at 6 months, p=0.30 baseline *versus* 6 months; **Supplementary Figure 2A**). It is important to note that during the follow up some patients were lost. Thus, it is not excluded that statistical significance could be achieved by increasing the number of patients. By contrast, in the *BRAF* mutated patient subgroup no difference were found in the PD-L1^+^ PMN frequency during immunotherapy (51.8% at baseline, 38.8% at 3 months; 54.5% at 6 months, p=0.87 baseline versus 6 months; **Supplementary Figure 2A**). We also investigated the impact of CD16^bright^CD62L^low^ PMN frequencies on survival in MP grouped according to the status of *BRAF* (mutated versus wild type). As shown in the **Supplementary Figure 2**, no difference in OS and PFS between MPs with high and low CD16^bright^CD62L^dim^ PMNs were found in *BRAF* wild type patient subgroup (**Supplementary Figures 2B, C**) nor in *BRAF* mutated patient subgroup (**Supplementary Figures 2D, E**). Taken together, these findings suggest that pre-treatment PD-L1^+^ PMN frequencies selectively predict patient clinical outcome and therapeutic response to nivolumab only in *BRAF* wild type melanoma patients.

However, the mechanisms underlying these findings remain unknown. Both ICIs and BRAF and MEK inhibitors can be effective in patients with *BRAF* ^V600E^ mutant melanoma. A subgroup analysis from CheckMate-067 demonstrated that the absolute difference in 5-year overall survival was substantially greater for the combination than nivolumab alone in patients with *BRAF*-mutant melanoma (60% ipilimumab plus nivolumab *vs.* 46% nivolumab alone). A smaller difference was observed for *BRAF* wild type (48% versus 43%) (3, 52). The long-term follow-up analysis of Checkmate 067 (6.5 year follow up analysis) confirmed the trend of continued separation between the combination and nivolumab monotherapy curves in patients with *BRAF* mutant disease (7). However, the study was not designed to formally compare these treatment groups or subgroups. Thus, despite *BRAF* mutated tumors, which can be efficiently treated with combination ICI therapy and/or targeted approaches, little progress has been made in identifying effective therapeutic options for the treatment of patients with wild-type *BRAF* melanomas (53). Our results demonstrate for the first time that a peripheral blood-derived biomarker (PD-L1^+^ PMN frequency) could independently predict patient clinical outcome to nivolumab in *BRAF* wild type melanoma patients, providing an additional tool for therapeutic choices in clinical oncology. Our study, however, does suffers from some limitations. Transcriptional characterization and functional assays on purified cells need to be performed, aimed at defining the identity of PMNs in these patients and their roles in melanoma progression and resistance to anti-PD-1 immunotherapy. In addition, the mechanisms responsible for the correlation observed between high PD-L1^+^ PMN frequency and *BRAF* status remains elusive. The expression of *BRAF* mutated in melanoma cells induced the up-regulation of IL-1α/β, which promoted the expression of PD-L1 in tumor-associated fibroblasts. These cells suppressed the activation and proliferation of melanoma-specific cytotoxic T cells, thus promoting an immunosuppressive TME (54). In murine models and human samples of *BRAF*-mutant melanoma, tumors induced the accumulation of regulatory T cells (Treg), which limited effector T cell activity (55). *BRAF*-mutated tumors did not only exhibit an enhanced infiltration by immunosuppressive immune cell populations but they also up-regulate the expression of genes associated with immunosuppression, such as CTLA-4, PD-L1, or HLA-G (55). *BRAF*-mutant human melanoma cell lines increased the expression of vascular endothelial growth factor (VEGF), which promoted a tolerogenic DC phenotype and tumor progression by sustaining neoplastic angiogenesis (55). Inhibition of BRAF^V600E^ in melanoma cell lines led to increased levels of melanocyte differentiation antigens (MDAs), which improved the recognition by antigen-specific T lymphocytes (56). Thus, the response to target therapy is not merely attributed to the direct effect of treatment on melanoma cells, but also to an immunomodulatory effect of therapy on TME and immune system activation. Thus, one could image that *BRAF* mutation in melanocytes could be responsible for a modulation of the TME which may overcome the predictive value of PD-L1 expression on peripheral blood neutrophils. Beyond *BRAF* mutations, various additional genomic abnormalities affecting additional genes, can drive melanoma initiation and progression, such as *NRAS, KIT, GNAQ, GNA11*, and *SF3B1*. The different molecular pathways responsible for the development and progression of melanomas are extremely complicated and interact with each other (via crosstalk mechanisms) to create resistance to treatment and the progression of cell signaling. Thus, in absence of *BRAF* mutations, alternative genomic abnormalities can be responsible for additional mechanisms of immuno-escape in our patient cohort, which could explain the role of PD-L1^+^ PMN frequencies in predicting clinical response to anti-PD-1 immunotherapy in the *BRAF* wild type MP subset. Finally, since stage III melanoma patients are currently considered candidates for anti-PD-1 therapy (4, 57), it would be necessary to assess whether PD-L1^+^ PMNs predict clinical outcomes in patients other than stage IV melanoma. To the best of our knowledge this is the first observation that peripheral blood MP PD-L1^+^ PMNs may have a value as predictive factor in *BRAF* wild type stage IV melanoma patients. Although these results need to be confirmed and validated in larger studies, they would suggest that prospectively PD-L1^+^ PMNs could be used as a circulating biomarker. Validation in an external cohort, which is in progress, may also aid in establishing the best cut-off reference for PD-L1^+^PMNs and its specificity for metastatic melanoma.

## Data availability statement

The raw data supporting the conclusions of this article will be made available by the authors, without undue reservation.

## Ethics statement

The studies involving human participants were reviewed and approved by Fondazione “G. Pascale” of Naples (prot. no 33/17) and of University of Naples Federico II (n. 301/12). The patients/participants provided their written informed consent to participate in this study.

## Author contributions

LC designed and conducted the experiments and drafted the manuscript. LF and VV recruited patients and provided clinical data. MC, GD’A, DM, and GM harvested clinical data and organized a database. LM, AF, SL, and GV provided technological support in ex vivo experiments. LC and DG performed statistical analysis. PA and MRG contributed to the experimental design, supervised the study, and revised the manuscript. All authors contributed to the article and approved the submitted version.

## Funding

This work was supported in part by grants from the CISI-Lab Project (University of Naples Federico II), TIMING Project and Campania Bioscience (Regione Campania) to MRG, SL, and GV and by MIUR-PRIN 2017M8YMR8_005 and AIRC under MFAG 2020 (grant number 25123) to MG.

## Acknowledgments

We would like to thank the patients who volunteered to participate in this study and the staff members who cared for them. We also thank Dr. Gjada Criscuolo for critical reading of the manuscript and the administrative staff (Dr. Roberto Bifulco, Dr. Anna Ferraro and Dr. Maria Cristina Fucci), without whom it would not be possible to work as a team.

## Conflict of interest

The authors declare that the research was conducted in the absence of any commercial or financial relationships that could be construed as a potential conflict of interest.

## Publisher’s note

All claims expressed in this article are solely those of the authors and do not necessarily represent those of their affiliated organizations, or those of the publisher, the editors and the reviewers. Any product that may be evaluated in this article, or claim that may be made by its manufacturer, is not guaranteed or endorsed by the publisher.

## Supplementary material

Supplementary Figure 1 Representative flow cytometric panels with respect to the gating strategy of total cells **(A)**, singlets **(B)**, live/CCR3- (**C**) and CCR3- CD66b+ CD11b+ PMNs **(D)**. **(E, F)** Representative flow cytometric panel with respect of the gating strategy of CD16^+^CD62^-^ cells in a healthy control **(E)** and in a melanoma patient **(F)**. Representative histograms illustrating cell counts for PD-L1 (red line) and FMO control (black line) in a healthy control **(G)** and in a melanoma patient **(H).**

Supplementary Figure 2
**(A)** Flow cytometry analysis of PD-L1^+^ live cells gated on CD66b^+^ CD11b^+^PMNs in MPs grouped according to BRAF status during nivolumab immunotherapy. Data were expressed as percentage of positive cells compared to FMO. The results were expressed as mean ± SD. **(B–E)** Kaplan–Meier survival curves show overall survival (OS %) **(B, D)** and progression-free survival (PFS %) **(C, E)** for advanced melanoma patients presenting a high (red line) or low (blue line) CD16^bright^CD62^low^ PMNs in *BRAF* wild-type **(B, C)**, and *BRAF* mutated **(D, E)** melanoma patients. Low and high CD16^bright^CD62^low^ PMN values were calculated using the median as the cut-off.

## Abbreviations

ICIs, immune checkpoint inhibitors; PMNs, polymorphonuclear leukocytes; TANs, tumor-associated neutrophils; MPs, melanoma patients; HCs, healthy controls; PFS, progression free survival; OS, overall survival; CR, complete response; PR, partial response; PD, progressive disease; SD, stable disease; HR, hazard ratio; ORR, overall response rate; DCR, disease control rate; TME, tumor microenvironment; CTLA-4, cytotoxic T lymphocyte-associated antigen 4; PD-1, programmed cell death protein 1; PD-L1, PD-1 ligand; FMO, Fluorescence Minus One; LDH, Lactate Dehydrogenase; ANC, Absolute Neutrophil Count.

## References

[1] Siegel RL Miller KD Jemal A. Cancer statistics, 2020. CA Cancer J Clin (2020) 70(1):7–30. doi: 10.3322/caac.21590 31912902

[2] Larkin J Chiarion-Sileni V Gonzalez R Grob JJ Cowey CL Lao CD. Combined nivolumab and ipilimumab or monotherapy in untreated melanoma. N Engl J Med (2015) 373(1):23–34. doi: 10.1056/NEJMoa1504030 26027431 PMC5698905

[3] Larkin J Chiarion-Sileni V Gonzalez R Grob JJ Rutkowski P Lao CD. Five-year survival with combined nivolumab and ipilimumab in advanced melanoma. N Engl J Med (2019) 381(16):1535–46. doi: 10.1056/NEJMoa1910836 31562797

[4] Eggermont AMM Blank CU Mandala M Long GV Atkinson V Dalle S. Adjuvant pembrolizumab versus placebo in resected stage III melanoma. N Engl J Med (2018) 378(19):1789–801. doi: 10.1056/NEJMoa1802357 29658430

[5] Robert C Grob JJ Stroyakovskiy D Karaszewska B Hauschild A Levchenko E. Five-year outcomes with dabrafenib plus trametinib in metastatic melanoma. N Engl J Med (2019) 381(7):626–36. doi: 10.1056/NEJMoa1904059 31166680

[6] Robert C Ribas A Schachter J Arance A Grob JJ Mortier L. Pembrolizumab versus ipilimumab in advanced melanoma (KEYNOTE-006): post-hoc 5-year results from an open-label, multicentre, randomised, controlled, phase 3 study. Lancet Oncol (2019) 20(9):1239–51. doi: 10.1016/S1470-2045(19)30388-2 31345627

[7] Wolchok JD Chiarion-Sileni V Gonzalez R Grob JJ Rutkowski P Lao CD. Long-term outcomes with nivolumab plus ipilimumab or nivolumab alone versus ipilimumab in patients with advanced melanoma. J Clin Oncol (2021) 40(2):127–37. doi: 10.1200/JCO.21.02229 PMC8718224 34818112

[8] Keir ME Butte MJ Freeman GJ Sharpe AH. PD-1 and its ligands in tolerance and immunity. Annu Rev Immunol (2008) 26:677–704. doi: 10.1146/annurev.immunol.26.021607.090331 18173375 PMC10637733

[9] Ribas A Hu-Lieskovan S. What does PD-L1 positive or negative mean? J Exp Med (2016) 213(13):2835–40. doi: 10.1084/jem.20161462 PMC5154949 27903604

[10] Burn GL Foti A Marsman G Patel DF Zychlinsky A. The neutrophil. Immunity (2021) 54(7):1377–91. doi: 10.1016/j.immuni.2021.06.006 34260886

[11] Sagiv JY Michaeli J Assi S Mishalian I Kisos H Levy L. Phenotypic diversity and plasticity in circulating neutrophil subpopulations in cancer. Cell Rep (2015) 10(4):562–73. doi: 10.1016/j.celrep.2014.12.039 25620698

[12] Granot Z Jablonska J. Distinct functions of neutrophil in cancer and its regulation. Mediators Inflamm (2015) 2015:701067. doi: 10.1155/2015/701067 26648665 PMC4663337

[13] Bingle L Brown NJ Lewis CE. The role of tumour-associated macrophages in tumour progression: implications for new anticancer therapies. J Pathol (2002) 196(3):254–65. doi: 10.1002/path.1027 11857487

[14] Qian BZ Pollard JW. Macrophage diversity enhances tumor progression and metastasis. Cell (2010) 141(1):39–51. doi: 10.1016/j.cell.2010.03.014 20371344 PMC4994190

[15] Galdiero MR Varricchi G Loffredo S Bellevicine C Lansione T Ferrara AL. Potential involvement of neutrophils in human thyroid cancer. PLoS One (2018) 13(6):e0199740. doi: 10.1371/journal.pone.0199740 29953504 PMC6023126

[16] Galdiero MR Bianchi P Grizzi F Di Caro G Basso G Ponzetta A. Occurrence and significance of tumor-associated neutrophils in patients with colorectal cancer. Int J Cancer (2016) 139(2):446–56. doi: 10.1002/ijc.30076 26939802

[17] Kargl J Zhu X Zhang H Yang GHY Friesen TJ Shipley M. Neutrophil content predicts lymphocyte depletion and anti-PD1 treatment failure in NSCLC. JCI Insight (2019) 4(24):1–16. doi: 10.1172/jci.insight.130850 PMC6975266 31852845

[18] Kim IS Gao Y Welte T Wang H Liu J Janghorban M. Immuno-subtyping of breast cancer reveals distinct myeloid cell profiles and immunotherapy resistance mechanisms. Nat Cell Biol (2019) 21(9):1113–26. doi: 10.1038/s41556-019-0373-7 PMC6726554 31451770

[19] Schalper KA Carleton M Zhou M Chen T Feng Y Huang SP. Elevated serum interleukin-8 is associated with enhanced intratumor neutrophils and reduced clinical benefit of immune-checkpoint inhibitors. Nat Med (2020) 26(5):688–92. doi: 10.1038/s41591-020-0856-x PMC8127102 32405062

[20] Bowers NL Helton ES Huijbregts RP Goepfert PA Heath SL Hel Z. Immune suppression by neutrophils in HIV-1 infection: role of PD-L1/PD-1 pathway. PloS Pathog (2014) 10(3):e1003993. doi: 10.1371/journal.ppat.1003993 24626392 PMC3953441

[21] Balch CM Gershenwald JE Soong SJ Thompson JF Atkins MB Byrd DR. Final version of 2009 AJCC melanoma staging and classification. J Clin Oncol (2009) 27(36):6199–206. doi: 10.1200/JCO.2009.23.4799 PMC2793035 19917835

[22] Eisenhauer EA Therasse P Bogaerts J Schwartz LH Sargent D Ford R. New response evaluation criteria in solid tumours: revised RECIST guideline (version 1.1). Eur J Cancer (2009) 45(2):228–47. doi: 10.1016/j.ejca.2008.10.026 19097774

[23] Muzio M Re F Sironi M Polentarutti N Minty A Caput D. Interleukin-13 induces the production of interleukin-1 receptor antagonist (IL-1ra) and the expression of the mRNA for the intracellular (keratinocyte) form of IL-1ra in human myelomonocytic cells. Blood (1994) 83(7):1738–43. doi: 10.1182/blood.V83.7.1738.1738 7908231

[24] Baumgarth N Roederer M. A practical approach to multicolor flow cytometry for immunophenotyping. J Immunol Methods (2000) 243(1-2):77–97. doi: 10.1016/S0022-1759(00)00229-5 10986408

[25] Borriello F Iannone R Di Somma S Loffredo S Scamardella E Galdiero MR. GM-CSF and IL-3 modulate human monocyte TNF-alpha production and renewal in *In vitro* models of trained immunity. Front Immunol (2016) 7:680. doi: 10.3389/fimmu.2016.00680 28138327 PMC5237654

[26] Schwartz LH Litiere S de Vries E Ford R Gwyther S Mandrekar S. RECIST 1.1-update and clarification: From the RECIST committee. Eur J Cancer (2016) 62:132–7. doi: 10.1016/j.ejca.2016.03.081 PMC5737828 27189322

[27] Gershenwald JE Scolyer RA. Melanoma staging: American joint committee on cancer (AJCC) 8th edition and beyond. Ann Surg Oncol (2018) 25(8):2105–10. doi: 10.1245/s10434-018-6513-7 29850954

[28] Stocks SC Ruchaud-Sparagano MH Kerr MA Grunert F Haslett C Dransfield I. CD66: role in the regulation of neutrophil effector function. Eur J Immunol (1996) 26(12):2924–32. doi: 10.1002/eji.1830261218 8977287

[29] Condliffe AM Chilvers ER Haslett C Dransfield I. Priming differentially regulates neutrophil adhesion molecule expression/function. Immunology (1996) 89(1):105–11. doi: 10.1046/j.1365-2567.1996.d01-711.x PMC1456672 8911147

[30] Pillay J Kamp VM van Hoffen E Visser T Tak T Lammers JW. A subset of neutrophils in human systemic inflammation inhibits T cell responses through mac-1. J Clin Invest (2012) 122(1):327–36. doi: 10.1172/JCI57990 PMC3248287 22156198

[31] Robert C Long GV Brady B Dutriaux C Maio M Mortier L. Nivolumab in previously untreated melanoma without BRAF mutation. N Engl J Med (2015) 372(4):320–30. doi: 10.1056/NEJMoa1412082 25399552

[32] Zhao Y Liu L Weng L. Comparisons of underlying mechanisms, clinical efficacy and safety between anti-PD-1 and anti-PD-L1 immunotherapy: The state-of-the-Art review and future perspectives. Front Pharmacol (2021) 12:714483. doi: 10.3389/fphar.2021.714483 34305619 PMC8293989

[33] Capone M Giannarelli D Mallardo D Madonna G Festino L Grimaldi AM. Baseline neutrophil-to-lymphocyte ratio (NLR) and derived NLR could predict overall survival in patients with advanced melanoma treated with nivolumab. J Immunother Cancer (2018) 6(1):74. doi: 10.1186/s40425-018-0383-1 30012216 PMC6048712

[34] Weide B Martens A Hassel JC Berking C Postow MA Bisschop K. Baseline biomarkers for outcome of melanoma patients treated with pembrolizumab. Clin Cancer Res (2016) 22(22):5487–96. doi: 10.1158/1078-0432.CCR-16-0127 PMC5572569 27185375

[35] Galdiero MR Varricchi G Loffredo S Mantovani A Marone G. Roles of neutrophils in cancer growth and progression. J Leukoc Biol (2018) 103(3):457–64. doi: 10.1002/JLB.3MR0717-292R 29345348

[36] Mei Z Shi L Wang B Yang J Xiao Z Du P. Prognostic role of pretreatment blood neutrophil-to-lymphocyte ratio in advanced cancer survivors: A systematic review and meta-analysis of 66 cohort studies. Cancer Treat Rev (2017) 58:1–13. doi: 10.1016/j.ctrv.2017.05.005 28602879

[37] Ferrucci PF Gandini S Battaglia A Alfieri S Di Giacomo AM Giannarelli D. Baseline neutrophil-to-lymphocyte ratio is associated with outcome of ipilimumab-treated metastatic melanoma patients. Br J Cancer (2015) 112(12):1904–10. doi: 10.1038/bjc.2015.180 PMC4580390 26010413

[38] Cassidy MR Wolchok RE Zheng J Panageas KS Wolchok JD Coit D. Neutrophil to lymphocyte ratio is associated with outcome during ipilimumab treatment. EBioMedicine (2017) 18:56–61. doi: 10.1016/j.ebiom.2017.03.029 28356222 PMC5405176

[39] Zaragoza J Caille A Beneton N Bens G Christiann F Maillard H. High neutrophil to lymphocyte ratio measured before starting ipilimumab treatment is associated with reduced overall survival in patients with melanoma. Br J Dermatol (2016) 174(1):146–51. doi: 10.1111/bjd.14155 26343230

[40] Ohtsu S Yagi H Nakamura M Ishii T Kayaba S Soga H. Enhanced neutrophilic granulopoiesis in rheumatoid arthritis. involvement of neutrophils in disease progression. J Rheumatol (2000) 27(6):1341–51. 10852252

[41] Manz MG Boettcher S. Emergency granulopoiesis. Nat Rev Immunol (2014) 14(5):302–14. doi: 10.1038/nri3660 24751955

[42] Scapini P Marini O Tecchio C Cassatella MA. Human neutrophils in the saga of cellular heterogeneity: insights and open questions. Immunol Rev (2016) 273(1):48–60. doi: 10.1111/imr.12448 27558327

[43] Hassani M Hellebrekers P Chen N van Aalst C Bongers S Hietbrink F. On the origin of low-density neutrophils. J Leukoc Biol (2020) 107(5):809–18. doi: 10.1002/JLB.5HR0120-459R PMC7318192 32170882

[44] Wang TT Zhao YL Peng LS Chen N Chen W Lv YP. Tumour-activated neutrophils in gastric cancer foster immune suppression and disease progression through GM-CSF-PD-L1 pathway. Gut (2017) 66(11):1900–11. doi: 10.1136/gutjnl-2016-313075 PMC5739867 28274999

[45] He G Zhang H Zhou J Wang B Chen Y Kong Y. Peritumoural neutrophils negatively regulate adaptive immunity *via* the PD-L1/PD-1 signalling pathway in hepatocellular carcinoma. J Exp Clin Cancer Res (2015) 34:141. doi: 10.1186/s13046-015-0256-0 26581194 PMC4652417

[46] Cheng Y Li H Deng Y Tai Y Zeng K Zhang Y. Cancer-associated fibroblasts induce PDL1+ neutrophils through the IL6-STAT3 pathway that foster immune suppression in hepatocellular carcinoma. Cell Death Dis (2018) 9(4):422. doi: 10.1038/s41419-018-0458-4 29556041 PMC5859264

[47] Zhou L Wang J Lyu SC Pan LC Shi XJ Du GS. PD-L1(+)NEUT, Foxp3(+)Treg, and NLR as new prognostic marker with low survival benefits value in hepatocellular carcinoma. Technol Cancer Res Treat (2021) 20:15330338211045820. doi: 10.1177/15330338211045820 34605709 PMC8493317

[48] Kwantwi LB Wang S Zhang W Peng W Cai Z Sheng Y. Tumor-associated neutrophils activated by tumor-derived CCL20 (C-c motif chemokine ligand 20) promote T cell immunosuppression *via* programmed death-ligand 1 (PD-L1) in breast cancer. Bioengineered (2021) 12(1):6996–7006. doi: 10.1080/21655979.2021.1977102 34519637 PMC8806641

[49] Hao S Andersen M Yu H. Detection of immune suppressive neutrophils in peripheral blood samples of cancer patients. Am J Blood Res (2013) 3(3):239–45. PMC3755524 23997986

[50] Podaza E Risnik D Colado A Elias E Almejun MB Fernandez Grecco H. Chronic lymphocytic leukemia cells increase neutrophils survival and promote their differentiation into CD16(high) CD62L(dim) immunosuppressive subset. Int J Cancer (2019) 144(5):1128–34. doi: 10.1002/ijc.31762 30178523

[51] Pisapia P Pepe F Iaccarino A Sgariglia R Nacchio M Russo G. BRAF: A two-faced janus. Cells (2020) 9(12):1–20. doi: 10.3390/cells9122549 PMC7760616 33260892

[52] Wolchok JD Chiarion-Sileni V Gonzalez R Rutkowski P Grob JJ Cowey CL. Overall survival with combined nivolumab and ipilimumab in advanced melanoma. N Engl J Med (2017) 377(14):1345–56. doi: 10.1056/NEJMoa1709684 PMC5706778 28889792

[53] Long GV Menzies AM Nagrial AM Haydu LE Hamilton AL Mann GJ. Prognostic and clinicopathologic associations of oncogenic BRAF in metastatic melanoma. J Clin Oncol (2011) 29(10):1239–46. doi: 10.1200/JCO.2010.32.4327 21343559

[54] Khalili JS Liu S Rodriguez-Cruz TG Whittington M Wardell S Liu C. Oncogenic BRAF(V600E) promotes stromal cell-mediated immunosuppression *via* induction of interleukin-1 in melanoma. Clin Cancer Res (2012) 18(19):5329–40. doi: 10.1158/1078-0432.CCR-12-1632 PMC3463754 22850568

[55] Jung T Haist M Kuske M Grabbe S Bros M. Immunomodulatory properties of BRAF and MEK inhibitors used for melanoma therapy-paradoxical ERK activation and beyond. Int J Mol Sci (2021) 22(18):1–16. doi: 10.3390/ijms22189890 PMC8469254 34576054

[56] Boni A Cogdill AP Dang P Udayakumar D Njauw CN Sloss CM. Selective BRAFV600E inhibition enhances T-cell recognition of melanoma without affecting lymphocyte function. Cancer Res (2010) 70(13):5213–9. doi: 10.1158/0008-5472.CAN-10-0118 20551059

[57] Weber J Mandala M Del Vecchio M Gogas HJ Arance AM Cowey CL. Adjuvant nivolumab versus ipilimumab in resected stage III or IV melanoma. N Engl J Med (2017) 377(19):1824–35. doi: 10.1056/NEJMoa1709030 28891423

