# Supplementary figures and images for: PD-L1+ neutrophils as novel biomarkers for stage IV melanoma patients treated with nivolumab
Source: Front Immunol. 2022 Aug 9;13:962669. doi: 10.3389/fimmu.2022.962669 (PMC9398490; doi:10.3389/fimmu.2022.962669)

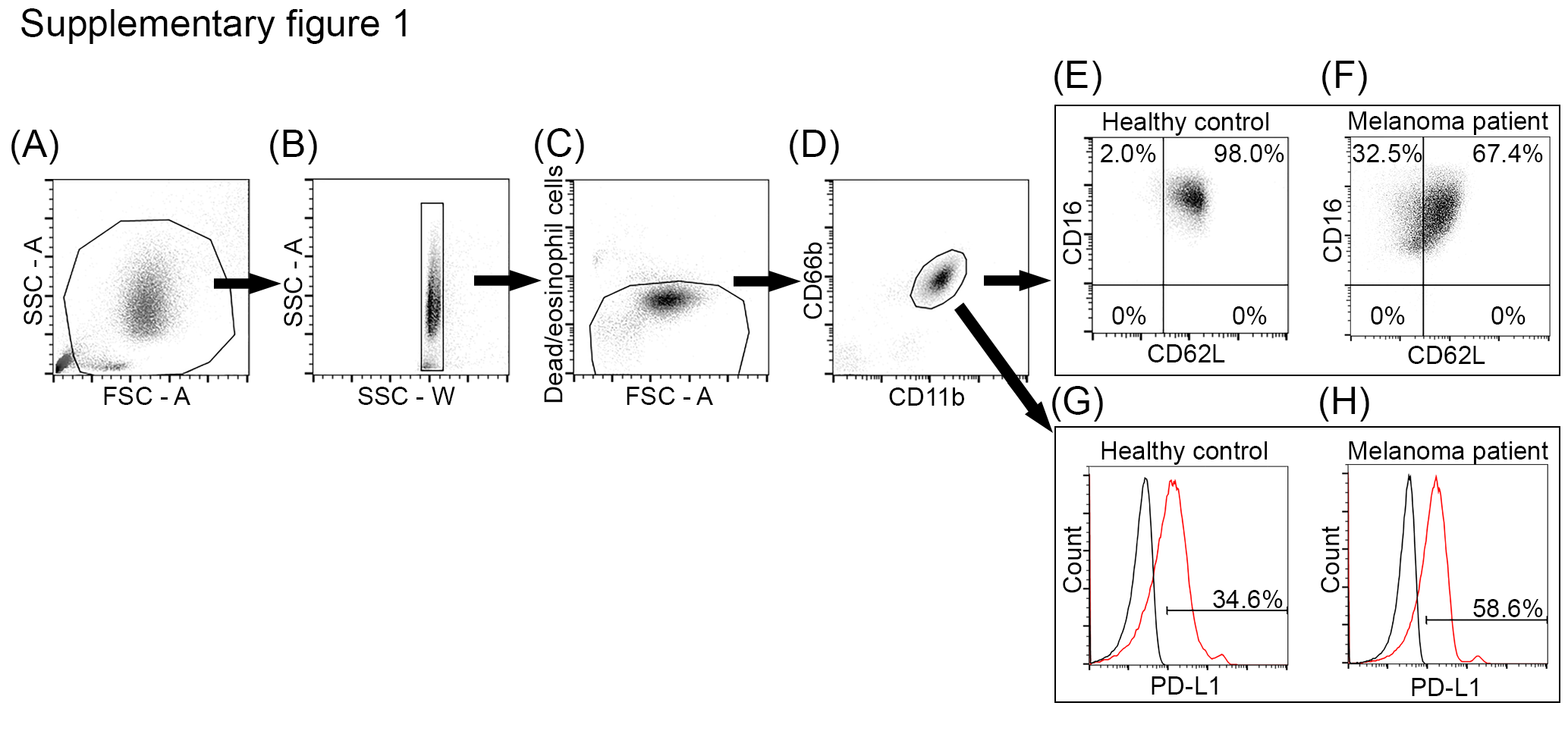

Supplement: Supplementary Figure 1 — Representative flow cytometric panels with respect to the gating strategy of total cells (A), singlets (B), live/CCR3- (C) and CCR3- CD66b+ CD11b+ PMNs (D). (E, F) Representative flow cytometric panel with respect of the gating strategy of CD16+CD62- cells in a healthy control (E) and in a melanoma patient (F). Representative histograms illustrating cell counts for PD-L1 (red line) and FMO control (black line) in a healthy control (G) and in a melanoma patient (H). [file Image_1.tif]

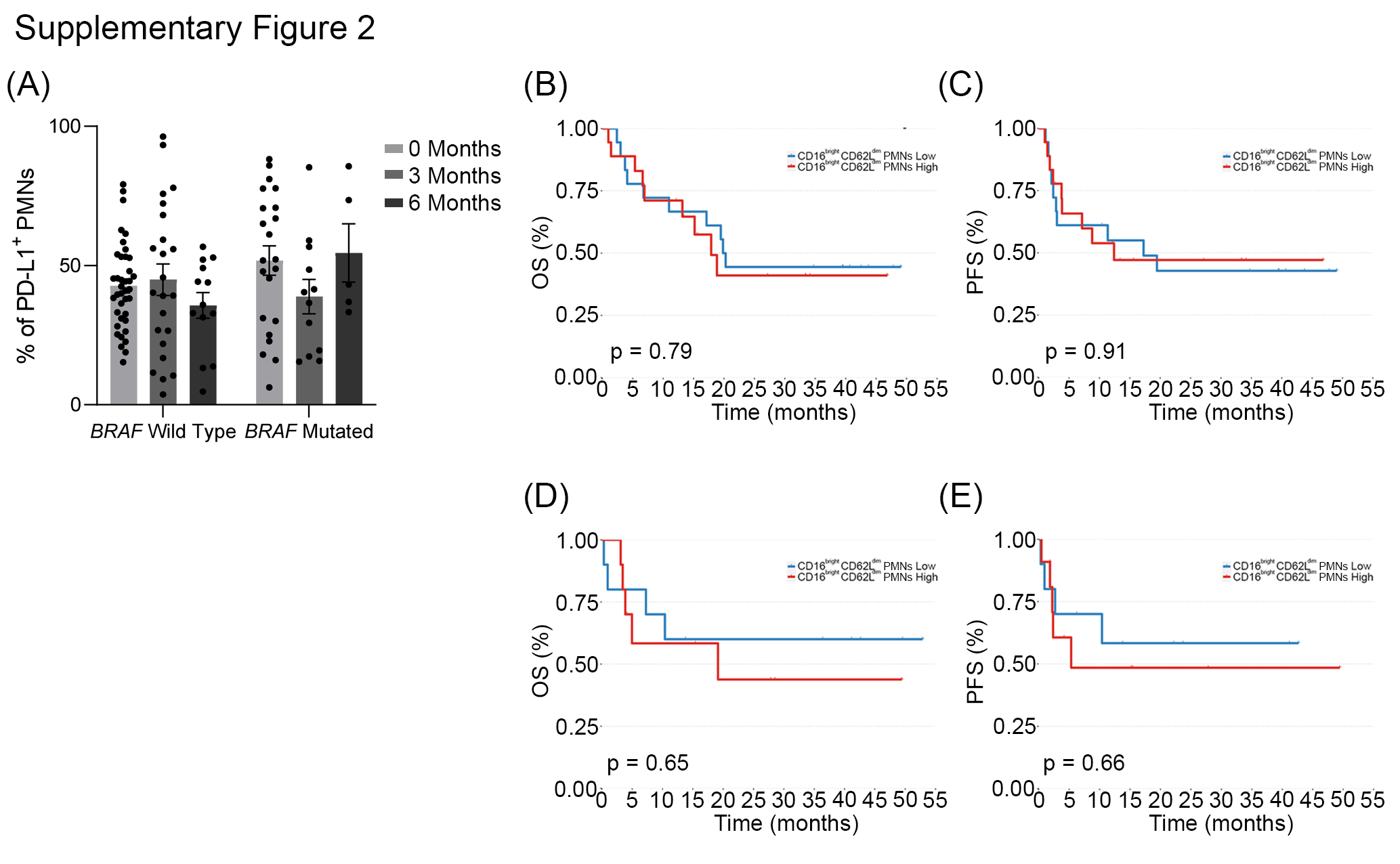

Supplement: Supplementary Figure 2 — (A) Flow cytometry analysis of PD-L1+ live cells gated on CD66b+ CD11b+PMNs in MPs grouped according to BRAF status during nivolumab immunotherapy. Data were expressed as percentage of positive cells compared to FMO. The results were expressed as mean ± SD. (B–E) Kaplan–Meier survival curves show overall survival (OS %) (B, D) and progression-free survival (PFS %) (C, E) for advanced melanoma patients presenting a high (red line) or low (blue line) CD16brightCD62low PMNs in BRAF wild-type (B, C), and BRAF mutated (D, E) melanoma patients. Low and high CD16brightCD62low PMN values were calculated using the median as the cut-off. [file Image_2.tif]
