# Supplementary material for: PD-L1+ neutrophils as novel biomarkers for stage IV melanoma patients treated with nivolumab
Source: Front Immunol. 2022 Aug 9;13:962669. doi: 10.3389/fimmu.2022.962669 (PMC9398490; doi:10.3389/fimmu.2022.962669)
Supplement: Supplementary file 3 [file Table_1.docx]

**Supplementary Table 1**. Demographic and clinical characteristics of MPs at baseline.

|  | ***Melanoma patients*** | | ***Healthy Controls*** | |
| --- | --- | --- | --- | --- |
|  | ***N*** | **%** | ***N*** | **%** |
| **Age** |  |  |  |  |
| Median, years**^#^** | 61 |  | 58 |  |
| Range | 21-87 |  | 26-75 |  |
| **Gender** |  |  |  |  |
| Male | 30 | 46.2 | 22 | 52.3 |
| Female | 35 | 53.8 | 20 | 47.6 |
| ***BRAF* mutation** |  |  |  |  |
| NO | 39 | 63.9 | NA | NA |
| YES | 22 | 36.1 | NA | NA |
| **Line of treatment** |  |  |  |  |
| 1 | 43 | 66.2 | NA | NA |
| 2 and 3 | 22 | 33.8 | NA | NA |
| **Distant metastasis** |  |  |  |  |
| M1a | 5 | 7.7 | NA | NA |
| M1b | 9 | 13.8 | NA | NA |
| M1c | 31 | 47.7 | NA | NA |
| M1d | 20 | 30.8 | NA | NA |
| **LDH** |  |  |  |  |
| Normal | 32 | 50.0 | NA | NA |
| Upper limit of normal | 32 | 50.0 | NA | NA |
| **ANC** |  |  |  |  |
| Median, n/μl**^#^** | 4000 |  | NA | NA |
| Range | 1500-18500 |  | NA | NA |

**N** number; **#**Age and ANC entered as continuous variables
