# Supplementary material for: PD-L1+ neutrophils as novel biomarkers for stage IV melanoma patients treated with nivolumab
Source: Front Immunol. 2022 Aug 9;13:962669. doi: 10.3389/fimmu.2022.962669 (PMC9398490; doi:10.3389/fimmu.2022.962669)
Supplement: Supplementary file 4 [file Table_2.docx]

**Supplementary Table 2.** Correlation between patients’ demographics and clinic pathologic features and PD-L1+PMNs

|  |  | **PMNs** | |  |
| --- | --- | --- | --- | --- |
|  | ***N*** | **PD-L1^+^ cells (%) *Mean (Std.Deviation)*** | ***p*** |  |
| **Age (years)** |  |  |  |  |
| < 61 | 32 | 49.11 (20.75) | 0.22 |  |
| ≥ 62 | 33 | 43.25 (17.57) |  |  |
| **Gender** |  |  |  |  |
| Male | 30 | 42.7 (15.87) | 0.19 |  |
| Female | 35 | 49.06 (21.58) |  |  |
| ***BRAF* mutation** |  |  |  |  |
| NO | 39 | 42.66 (15.42) | 0.08 |  |
| YES | 22 | 51.84 (24.77) |  |  |
| **Line of treatment** |  |  |  |  |
| 1 | 43 | 43.86 (17.87) | 0.19 |  |
| 2 and 3 | 22 | 50.57 (21.52) |  |  |
| **Distant metastasis** |  |  |  |  |
| M1a | 5 | 34.08 (13.11) | 0.38***** |  |
| M1b | 9 | 43.32 (15.31) |  |  |
| M1c | 31 | 49.47 (21.15) |  |  |
| M1d | 20 | 45.23 (18.70) |  |  |
| **LDH** |  |  |  |  |
| Normal | 32 | 48.23 (22.15) | 0.44 |  |
| Upper limit of normal | 32 | 44.45 (16.23) |  |  |
| **ANC** |  |  |  |  |
| <4000 | 28 | 47.17 (19.08) | 0.86 |  |
| ≥4000 | 30 | 46.23 (20.09) |  |  |

Student t test; *****One-way ANOVA and Sidak's multiple comparisons test. **PMNs** polymorphonuclear cells; **N** number.
